# Editorial: Source Imaging in Drug Resistant Epilepsy - Current Evidence and Practice

**DOI:** 10.3389/fneur.2020.00056

**Published:** 2020-02-11

**Authors:** Sándor Beniczky, Eugen Trinka

**Affiliations:** ^1^Department of Clinical Neurophysiology, Danish Epilepsy Centre, Dianalund, Denmark; ^2^Department of Clinical Neurophysiology, Aarhus University Hospital, Aarhus, Denmark; ^3^Department of Clinical Medicine, Aarhus University, Aarhus, Denmark; ^4^Department of Neurology, Christian Doppler Clinic, Paracelsus Medical University, Salzburg, Austria; ^5^Center for Cognitive Neuroscience, Salzburg, Austria

**Keywords:** EEG, epilepsy, ictal, interictal, MEG, presurgical evaluation, source imaging, source analysis

Localizing the source of electroencephalography (EEG) and magnetoencephalography (MEG) signals has been the objective of extensive research in the last decades. Imaging the source of epileptiform activity in the brain is especially important for patients with drug-resistant focal epilepsy, since it provides clinically useful information for planning the surgical therapy.

For long time, source imaging (SI) has been considered an experimental technique. A recent survey published by the E-PILEPSY consortium, comprising 25 European centers, showed that less than half of the centers used these methods for presurgical evaluation (1). There are multiple possible causes for the under-utilization of this method. Many clinicians are skeptical about SI because they are not aware of the evidence provided by numerous clinical trials. Often, clinicians doing presurgical evaluation lack the expertise in advanced signal analysis.

To address this problem and to facilitate clinical implementation of SI in the presurgical evaluation of patients with drug-resistant focal epilepsy, in this special issue (eCollection) of Frontiers in Neurology, we present a series of papers that provide evidence for the accuracy of SI, explain the technical background in a language accessible for clinicians and emphasize the advantages and the limitations of this method.

Sharma et al. present the results of a large meta-analysis of EEG and MEG SI, based on data from 1,152 operated patients. They found that these methods have high sensitivity (up to 90%) and diagnostic odds ratio (up to 7.9).

Scherg et al. explain the basic principles of EEG signal generation, and how to take the EEG recorded from scalp sensors back into the brain. They describe a novel method of visualizing the signals in the source space, by using the power of multiple discrete sources.

The practical review by Michel and Brunet explains these different steps in SI. The authors illustrate the process of SI, in a comprehensive analysis pipeline using a stand-alone freely available academic software.

Carrette and Stefan review the current practice for using magnetic SI of focal interictal and ictal epileptic activity during the presurgical evaluation of drug resistant patients.

Besides localization of the epileptic focus (what needs to be resected), in presurgical evaluation it is important to localize the eloquent cortex (what must not be resected). Kreidenhuber et al. provide a general overview of MEG and high-density EEG based methods of functional cortical mapping.

van Mierlo et al. describe the potential of EEG and MEG source connectivity to provide an intuitive view of the epileptic activity in the brain, that help localizing the seizure onset zone and the irritative zone.

High-frequency oscillations (HFOs) are promising biomarker of the epileptic focus. Most of the evidence is still based on invasive recordings; nevertheless, there is increasing expertise with recording HFOs non-invasively. Thomschewski et al. review the current literature on this topic, with emphasis on findings and technical considerations regarding their localization.

SI is an important tool in the presurgical evaluation of patients with drug-resistant focal epilepsy. It provides non-redundant, clinically useful information. The papers in this special issue (eCollection) of Frontiers in Neurology summarize the published evidence on the accuracy of various SI methods. The papers in this eCollection help the readers to integrate this method into their presurgical workup, but in the same time, they emphasize the current limitations of SI and propose further development of the methods, especially for automatizing the analysis and extending the method to imaging of connectivity changes in these patients.

## Author Contributions

SB drafted the manuscript. All authors contributed to editing the manuscript.

### Conflict of Interest

The authors declare that the research was conducted in the absence of any commercial or financial relationships that could be construed as a potential conflict of interest.
